# The Influence of Human-Organizational Factors on Falling Accidents From Historical Text Data

**DOI:** 10.3389/fpubh.2021.783537

**Published:** 2022-01-11

**Authors:** Xixi Luo, Quanlong Liu, Zunxiang Qiu

**Affiliations:** School of Economics and Management, China University of Mining and Technology, Xuzhou, China

**Keywords:** falling accidents, human-organizational factors, human factor analysis and classification system (HFACS), Bayesian network, fuzzy set theory

## Abstract

This paper firstly proposes a modified human factor classification analysis system (HFACS) framework based on literature analysis and the characteristics of falling accidents in construction. Second, a Bayesian network (BN) topology is constructed based on the dependence between human factors and organizational factors, and the probability distribution of the human-organizational factors in a BN risk assessment model is calculated based on falling accident reports and fuzzy set theory. Finally, the sensitivity of the causal factors is determined. The results show that 1) the most important reason for falling accidents is unsafe on-site supervision. 2) There are significant factors that influence falling accidents at different levels in the proposed model, including operation violations in the unsafe acts layer, factors related to an adverse technological environment for the unsafe acts layer, loopholes in site management in the unsafe on-site supervision layer, lack of safety culture in the adverse organizational influence layer, and lax government regulation in the adverse external environment layer. 3) According to the results of the BN risk assessment model, the most likely causes are loopholes in site management work, lack of safety culture, insufficient safety inspections and acceptance, vulnerable process management and operation violations.

## Introduction

To improve the legal system for emergency management, standardize the investigation and handling of production safety accidents, and protect people's lives and property, China issued the “Regulations on Reporting and Investigation and Handling of Production Safety Accidents,” which clearly proposes measures to improve accident investigation mechanisms and the preparation of accident investigation reports. These measures can be applied to assess responsibility and learn from accidents over time, which is conducive to promoting the implementation of safe production measures (1). In recent years, the accident rate in the construction industry has been the highest among all industrial production sectors, and the safety of construction workers has become a global problem (2, 3). According to statistics from the Ministry of Housing and Urban-Rural Development in China, 7,878 construction safety accidents occurred across the country between 2007 and 2019, resulting in 9,548 deaths (4); these accidents not only generated vast economic losses but also seriously affected societal stability. Among the reported construction accidents, falling from heights was most common for 54.57% of all accidents. Thus, the occurrence of safety accidents can be potentially limited by studying falling accidents in construction and implementing corresponding measures. In terms of the causes of falling accidents, Martín et al. (5) suggested that many factors can cause falls in the construction process, but a maximum of 90% of accidents are caused by human-related factors. It is important to reduce the occurrence of falling accidents, identify the human factors that contribute to falling accidents and propose corresponding improvement measures according to the key influential factors.

An increasing number of high-risk industries have gradually realized that human factors and organizational factors are the main causes of accidents (6), and many scholars have conducted research on the definition of human-organizational factors, the mechanisms of accidents and influencing factors, the methods of analyzing human error and other related topics. For example, Leveson (7) proposed the system-theoretic accident modeling and processes (STAMP) method, which emphasizes not only the identification of accident causes from the perspective of complex social technology systems but also includes coupled and interactive factors, such as human factors and organizational factors. Daramola (8) utilized the human factor analysis and classification system (HFACS), which is a human factor research tool based on system theory, to analyse human error factors related to safety accidents. Hollnagel (9) proposed the cognitive reliability and error analysis method (CREAM), emphasizing the important influence of the situational environment on human behavior; the unique cognitive model provides root cause traceability and human error probability prediction. There are many methods and tools for human factor analysis in different fields, among which HFACS is currently one of the most widely applied tools. Comprehensive and effective accident analysis results have sparked significant research attention on HFACs in various industrial fields (10, 11). Although the HFACS framework provides a reliable tool for the identification and analysis of human-organizational factors related to safety accidents, there are limitations in specific research applications due to the lack of quantitative analysis and the unclear causal relationships among research factors. To enhance the ability of the HFACS to assess human factors in detail during the process of accident investigation, many studies have combined quantitative analysis with the HFACS framework. For example, the HFACS framework has been combined with the analytical network process (ANP) method (12), fuzzy Bayesian network (FBN) method (13), structural equation model (SEM) method (14) and other quantitative research theories. Notably, BNs are considered the most effective for analyzing the dependence among factors in an uncertain research environment and are widely employed in the field of security.

There have been many studies of the causative factors of construction accidents, but few studies have conducted human factor analysis based on actual historical accident reports. The non-reproducibility of construction accidents determines that the investigation of accident causes mainly depends on interviews and sensing information. Therefore, the investigation of accident causes has strong uncertainty and subjectivity. How to improve the accuracy of accident cause investigations through relevant information after the occurrence of accidents has become a very important research topic. Therefore, this paper, first, revises the original HFACS framework according to the characteristics of falling accidents and establishes a risk assessment model for falling accidents based on interfactor dependence. Second, the collected falling accident reports and fuzzy set theory were combined to infer a BN. Finally, the probability distribution and human-organizational sensitivity factors in falling accidents are calculated to identify the potential causes of falling accidents in construction and to provide theoretical guidance for impact mechanism analysis, safety risk prevention, and accident report rectification related to falling accidents.

## Materials and Methods

### Research Methods

#### Human Factors Analysis and Classification System (HFACS)

The HFACS was proposed by Shappell and Wiegmann (15); its development was inspired by the “Swiss cheese” model of Reason (16). The HFACS is currently widely employed in human factor analyses of safety accidents based on system theory. The “Swiss cheese” model proposed by Reason divides the causes of accidents into four levels and visually compares errors to “holes” in the systems corresponding to different levels. When errors at all levels yield risks that break through the defence line, a safety accident occurs. Based on the “Swiss cheese” model, the HFACS method can be utilized to determine the causes of accidents at different levels, and the human-organizational factors of safety accidents can be comprehensively and systematically analyzed. Notably, the causes of accidents can be traced, and targeted safety precautions can be established at all levels.

The research related to the HFACS has mainly supplemented the framework content and expanded HFACS applications. Specifically, the original HFACS framework has four levels of human error: adverse organizational influences, unsafe on-site supervision, preconditions for unsafe acts, and unsafe acts from top to bottom. With the development of accident cause theory, research on human factors at the government and environmental levels has gradually received attention. Reinach and Viale (17) added “outside factors” to the HFACS framework for the railway field, which classified causes into “regulatory oversight” and “other” categories. When Chen et al. (18) investigated the human-organizational causes of maritime accidents, a fifth layer that included external factors and “legislation gap,” “administrative oversight,” and “design flaw” categories in combination with the International Maritime Organization (IMO) guidelines, was added. In view of the increasing emphasis on external factors such as the economy and environment, the identification of potential influential factors with the HFACS model must be supplemented and improved according to actual cases. In the expansion of the HFACS research scope, Shappell and Wiegmann (19) initially developed an HFACS model for safety analyses of military aviation accidents. Similar models have been applied in shipping (20), coal mine (21), chemical industry (22), railway (23), and construction (24) research, as shown in Table 1. Although it has been widely applied in different fields, the HFACS model has rarely been applied in studies of the construction industry, potentially due to the insufficient attention given to the human- and organization-based causative factors associated with safety accidents in the construction industry.

**Table 1:** List of HFACSs in continuous improvement in different industries.

**Literature sources**	**Industry**	**HFACS version**	**Key modifications**	**Main findings**
Daramola (8)	Aviation industry	Modified- HFACS	The improved framework was more suitable for the analysis of human factors related to civil aviation accidents, and the technical environment was added to the second layer.	Findings from the research highlight the need to address personnel skills, physical environment issues (mostly weather-related) and supervisory competence.
Wrobel et al. (43)	Shipping industry	HFACS-MA	The addition of a fifth level called external influence includes administration oversights, design flaws and legislation gaps. In the second level, the impact of software and hardware on the safety performance has been added.	Implementation of unmanned ships might reduce the number of navigation-related accidents like collisions or groundings.
Kaptan et al. (44)	Shipping industry	HFACS-PV	The addition of a fifth level called operational conditions includes internal conditions and external conditions.	Unqualified crew assignment and lack of training and familiarization were found to be the most critical factors.
Verma and Chaudhari (45)	Coal mine industry	Modified- HFACS	The addition of a fifth level called outside factors with the factors of regulatory factors and other.	Skill-based errors are most critical and require immediate attention for mitigation.
Liu et al. (14)	Coal mine industry	HFACS-CM	The addition of a fifth level called external environment includes management factors, political factors, economic factors and historical factors.	From the most impactful factor to the least impactful factor are external environment, unsafe leadership, preconditions for unsafe acts, and organizational influences.
Xia et al. (33)	Chemical industry	Modified- HFACS	The addition of a fifth level called emergency failure includes emergency resource errors; not timely emergency; inappropriate emergency.	The individual level human factors should be managed from the perspectives of safety skills, work attitude and personal health status.
Wang et al. (46)	Chemical industry	HFACS-CSME	The definition of each cause factor in the original model was retained and supplemented with corresponding specific manifestations.	Based on the further revision of manifestations and causes classification, a new model consisting of 15 cause factors and 56 manifestation forms was obtained.
Zhan et al. (47)	Railway industry	HFACS-RAs	The accident casual factors in the second level are further changed to Substandard Conditions of Operators, Substandard Conditions of Team, Adverse Conditions of Mission and Adverse Physical Environment.	The critical problem existing in organization level indirectly such as insufficient training quality and management.
Hale et al. (48)	Construction industry	Modified- HFACS	Combine the content of the third and fourth levels and increase a fifth level called environmental influences, including political, regulatory, market and social influences.	The underlying factors associated with inadequacies in planning and risk assessment, competence assurance, hardware design, purchase and installation, and contracting strategy.
Ye et al. (49)	Construction industry	I-HFACS	The addition of a fifth level with the two categories called regulatory factors and economic/political/social/legal environment. In level 4, the factors of organizational climate were replaced with safety culture.	Seven key factors were regulatory factors, organizational process, supervisory violations, adverse spiritual state, skill underutilization, skill-based errors, and violations.

#### Bayesian Networks

A BN is a tool that combines probability theory with graph theory to perform uncertainty reasoning and data analysis in complex fields; specifically, a visual network graph is used to visualize the probability relationships among variables. The composition of a BN is divided into qualitative parts and quantitative parts. At the qualitative level, a directed acyclic graph (DAG) is used to represent the dependent and independent relationships between two variable sets, and at the quantitative level, a conditional probability table (CPT) is utilized to describe the dependent relationships among variables and their parent nodes (25). A BN can be defined as *N* = < *G, P* >, where *G* is the structure diagram of the BN, *G* = < *V, E* >, *V* represents the set of nodes *V*_1_, *V*_2_, …, *V*_*n*_, and each node represents different random events. There are three types of nodes in BNs: target node (leaf node), evidence node (parent node) and intermediate node (child node). *E* represents the set of directed edges with dependencies between two nodes, usually from the parent node to the child node. *P* represents the parameter set of the BN, including the prior probability table and CPT of nodes, which are utilized to represent the dependency strength between two nodes. The prior probability can be learned from prior knowledge or data, and the conditional probability distribution of each variable (*X*_*i*_) is based on its parent node. The parameter is expressed as *p*(*X*_*i*_|π(*X*_*i*_)), where π(*X*_*i*_) is the parent set of the variable *X*_*i*_. Semantically, a BN represents the union of the CPTs of all nodes. By decomposing the joint probability distribution, the complexity of the probability calculation process is reduced. *Via* the independent and dependent relationships among variables, a BN provides predictions and solutions for uncertain problems. The essence of BN calculations is to optimize the relevant parameters by determining the prior and posterior probabilities for a specific network structure.

#### Fuzzy Bayesian Networks

Existing construction accident reports focus on identifying the responsible parties of an accident. The in-depth, human factor investigation of an accident has a strong uncertainty, and available historical data are limited, so it is difficult to express the probability of an event with a definite numerical value. However, BN analysis that is based on fuzzy theory is suitable for modeling research in the new field of uncertainty. Therefore, BNs and fuzzy set theory can be combined to construct FBNs, which can be employed for the quantitative treatment of boundary uncertainty and uncertainty problems at nodes (26, 27). The steps involved in applying a BN for safety accident risk assessment can be divided into network topology establishment, probability determination of node parameters, network learning and reasoning, risk assessment and sensitivity analysis.

1) The establishment of a network topology refers to the formation of a network association structure based on real accident scenes and the relationships among security risks and accidents. The following factors need to be considered in this process: ①factor identification and status determination, ②logic structure combination, and BN transformation.

2) Probability determination for node parameters refers to measuring the prior probability and conditional probability of each node in the BN based on statistics or expert consultation. Many data samples need to be collected when node parameters are calculated based on statistical methods these samples are obtained according to probability theory and a Bayesian formula. If the data sample size is insufficient, the expert consultation method combined with fuzzy set theory is generally adopted to obtain the prior probability distribution table for evidence nodes (without parent nodes) and conditional probability distribution table for intermediate nodes.

①Bayesian parameter estimation with complete data

After construction of the BN topology, the maximum likelihood estimation (MLE), maximum a posteriori (MAP) estimation, Bayesian estimation and empirical Bayesian (EB) can be applied for parameter learning to determine the conditional probability distribution among the relevant variables (28). MLE is generally applicable to the parameter estimation of large sample sizes, and the estimated value shows agreement with the actual value. In this paper, a large number of construction falling accident report texts can be collected, so MLE can be utilized for parameter learning. Conceptually, MLE uses parameter θ to calculate the value with the highest fitting degree for data set *D* (29). The calculation process is expressed as follows:

Step 1: Construct the likelihood function for θ:$L\left(\theta \right)=\underset{i=1}{\overset{n}{\Pi }}P\left(X_{i}|\theta \right)$

Step 2: Take the logarithm of *L*(θ), and obtain the derivative:$\frac{d\ln\;L}{d\theta }=0$

Step 3: Solve the likelihood function and obtain the MLE value θ^*^ for parameter θ.

where *X*_*i*_ is the state value of the dataset *D*, θ is the parameter to be estimated, and *P*(*X*_*i*_|θ) is the conditional probability of variable *X*_*i*_ based on parameter θ.

②Bayesian parameter estimation in the case of missing data

To overcome issues related to uncertainty and a lack of sufficient data support, the concept of “linguistic variables” can be considered in semantic and probability conversion (30). Based on a fuzzy semantic probability table, experts give fuzzy scores for different risk states; then, by processing the obtained data, the BN parameter values for the target nodes are obtained. In this study, seven language terms are used to estimate the probability of occurrence of basic events, and each fuzzy number is processed according to a triangular fuzzy function. The results are shown in Table 2.

**Table 2:** Semantic terms and corresponding triangular fuzzy numbers.

**Number**	**Semantic term**	**Triangular fuzzy numbers**
1	Certain	(0.85,1,1)
2	Probable	(0.75,0.85,1)
3	Expected	(0.5,0.75,0.85)
4	Fifty-fifty	(0.25,0.5,0.75)
5	Uncertain	(0.15,0.25,0.5)
6	Improbable	(0,0.15,0.25)
7	Impossible	(0,0,0.15)

The probabilities of the parameters can be obtained by averaging, defuzzifying and normalizing the fuzzy probabilities. Firstly, the fuzzy probabilities obtained from different experts are arithmetically averaged, and the formula is expressed as follows:


(1) $$\begin{array}{r} P_{ij}=\frac{{P_{ij}}^{1}+{P_{ij}}^{2}+\cdots +{P_{ij}}^{m}}{m}=\left(a_{ij},n_{ij},b_{ij}\right) \end{array}$$

where *P*_*ij*_ is the fuzzy probability that the state of the *i*-th node is *j*; ${P_{ij}}^{m}$ is the fuzzy probability given by the *m*-th expert for the *i*-th node status of *j*; and (*a*_*ij*_, *n*_*ij*_, *b*_*ij*_) are the parameters of the triangular fuzzy function.

Then, the probability of occurrence for different events is converted into a precise value through the process of defuzzifying. In this paper, the “mean area method” is utilized in the defuzzifying process, and the formula is expressed as follows:


(2) $$\begin{array}{r} {P_{ij}}^{{}^{\prime }}=\frac{a_{ij}+2n_{ij}+b_{ij}}{4} \end{array}$$

Finally, the accurate probability values of the nodes in different risk states are normalized, and the sum of the probabilities for the same node in different states is 1. The corresponding process can be expressed as follows:


(3) $$\begin{array}{c} \bar{P_{ij}}=P_{ij}{}^{{}^{\prime }}/\overset{k}{\underset{j=0}{\sum^{\text{​}}}}P_{ij}{}^{{}^{\prime }} \end{array}$$

3) Network learning and reasoning

Network learning and reasoning involve calculating the probability of a target node based on the prior probability table and CPT associated with a known node. According to the different directions of reasoning and the roles of node variables, the Bayesian reasoning process can be divided into two modes. The first mode is 1) positive causal reasoning, that is, the reasoning process from cause to effect. Given the probability values of all root nodes at different risk levels, the BN topology and parameter values are combined to obtain the probability results. The second mode is 2) reverse diagnostic reasoning, that is, the reasoning process from result to cause. Given the probability values of the target node at different risk levels, the probability of each cause can be determined for a given event.

4) Risk assessment and sensitivity analysis

Risk assessment refers to the identification of accident risk levels and key causal factors under different conditions based on the reasoning process. In a positive causal reasoning network, the grade corresponding to the maximum probability value in the target probability distribution is selected as the risk probability grade, and the key causative factors of accidents can be determined by combining this approach with a reverse diagnostic reasoning network. Sensitivity analysis involves identifying the factors that have the greatest impact on the occurrence of accidents and quantifying them considering the degree of influence and the parameters of the target node (31). The sensitivity factor is denoted as α and expressed as follows for the *i*-th basic event:


(4) $$\begin{array}{r} \alpha_{i}=\frac{\left(P^{T}-{P^{T}}_{i}\right)/P^{T}}{\max\left\{\left(P^{T}-{P^{T}}_{i}\right)/P^{T}\right\}} \end{array}$$

where *P*^*T*^ is the probability of a risk event; ${P^{T}}_{i}$ is the probability of a risk event when the *i*-th basic event does not occur; and *i* = 1, 2, …, *m*.

### Data Sources

This paper mainly collects safety accident reports through network screening to obtain original data related to the human-organizational causative factors of falling accidents in construction. Safety accident reports were mainly collected from the Ministry of Housing and Urban-Rural Development, State Administration of Production Safety Supervision and Management, websites of various administrative departments, safety management network, municipal governments and various safety supervision bureaus. A total of 432 reports of construction falling accidents in China from 29 to 29 were collected (32), focusing on the major production safety accident reports and general production safety accident reports. The cases judged as non-liability accident or near misses were excluded from the analysis. In addition, the text of the accident report mainly included the following four aspects: general situation of the accident unit, process of the accident, casualties and direct economic losses caused by the accident, causes and nature of the accident, and identification of the accident responsibility.

## Research Framework

**Figure 1:**
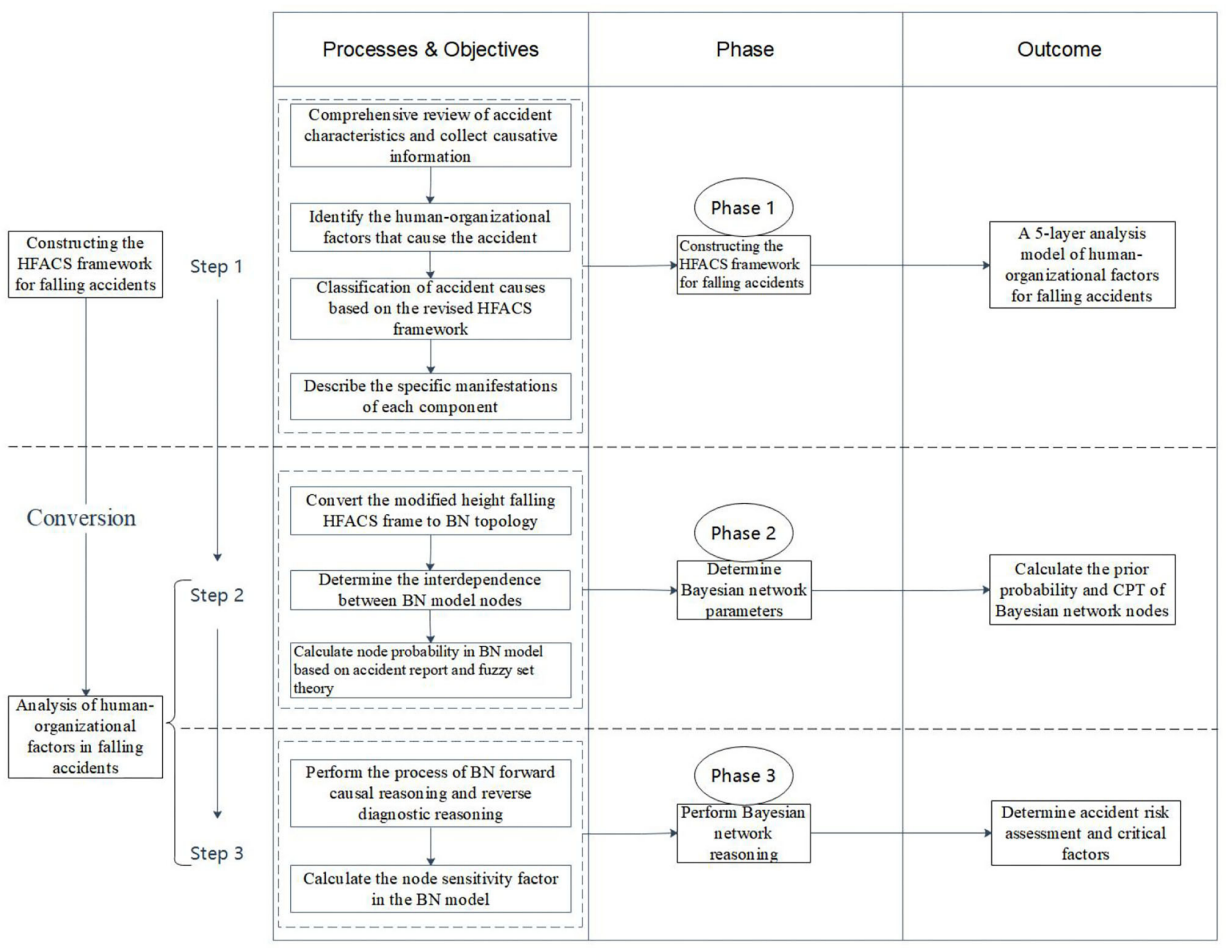
Research framework.

To clearly describe the overall research process of the human-organizational factor analysis of falling accidents by integrating the HFACS and BN methods, the research framework is shown in Figure 1.

## Revised Design of the Hfacs Framework for Falling Accidents in Construction

### Constructing the Corrected HFACS Framework for Falling Accidents

**Figure 2:**
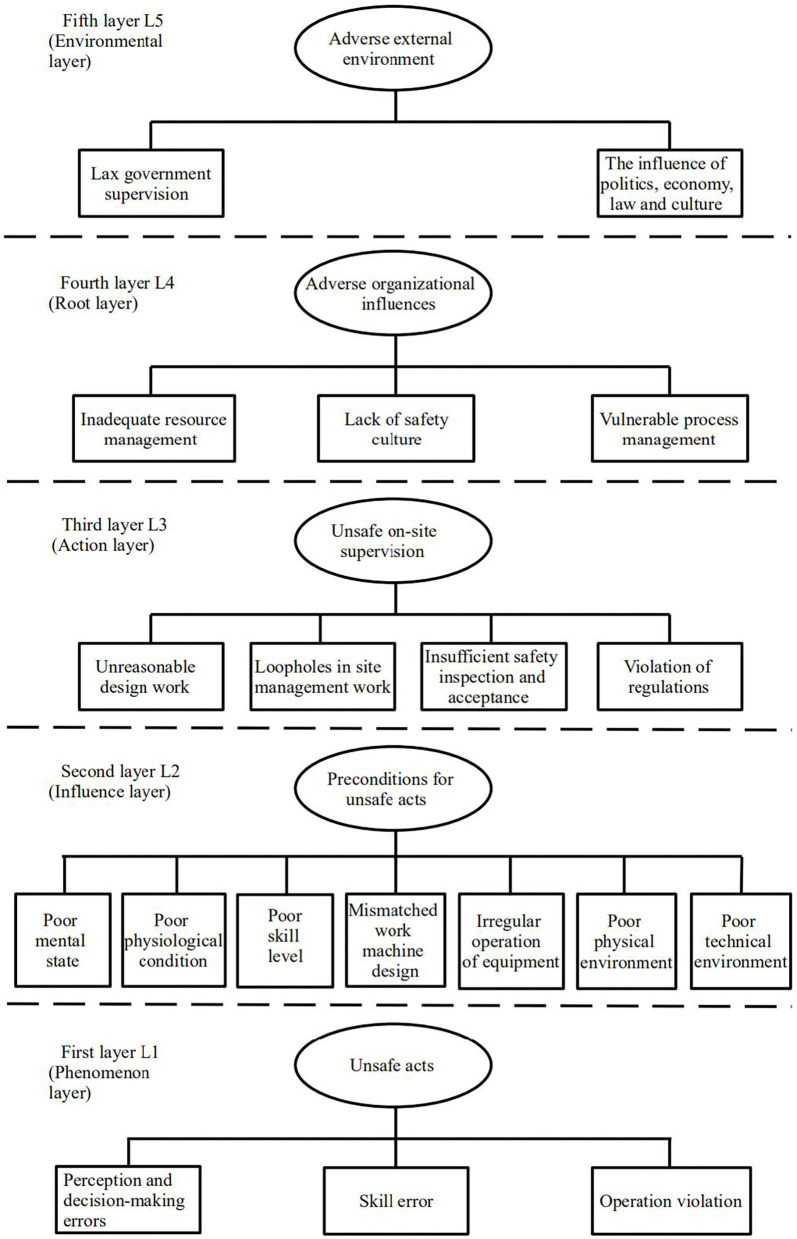
HFACS framework for falling accidents in construction.

The original HFACS framework was proposed for human factor analyses of aviation accidents without considering the influence of external factors. As the application of HFACS has gradually expanded, it has become necessary to modify the general model accordingly. Garrett and Teizer applied the HFACS in the construction industry for the first time in conjunction with the human error awareness training (HEAT) model to investigate the causes of human errors in construction. Xia et al. (33) adjusted the HFACS framework according to the specific characteristics of the construction industry to effectively analyse construction safety performance. In addition to adding to and deleting some of the original components, external environmental impact layers, including stakeholders, social and industrial environments, legislation and enforcement, were added. In this paper, the HFACS framework is applied to falling accidents in building construction. Based on the collected accident cases and an analysis of the existing literature, the original HFACS framework is modified, and an HFACS framework suitable for falling accidents in building construction is proposed, as shown in Figure 2.

#### L1: Unsafe Acts

Reason proposed that in analyses of safety accidents, human factors can be divided into errors and violations. Errors indicate that a person's ability or psychological level did not achieve the expected results. Zhang and Fang (34) proposed that errors can be divided into cognitive, decision-making and skill errors. According to the different forms of deviation, decision-making errors will occur when the available information and knowledge is insufficient; cognitive errors will occur when the concept of personal safety is not prioritized or does not conform to realistic requirements; and skill errors will occur when individuals who engage in the work do not understand or remember the skills required. A violation is a deliberate deviation from the defined safety rules and operating procedures, and violations can be habitual or accidental (35). For example, individuals working at high altitudes need to wear protective equipment correctly, but some personnel deliberately do not wear or improperly wear protective equipment during climbing, thus creating a potential safety hazard.

In summary, the unsafe acts of construction workers are influenced by three factors: perception and decision-making errors (L1R1), skill errors (L1R2), and operational violations (L1R3).

#### L2: Preconditions for Unsafe Acts

Liang (36) noted that unsafe acts are usually the result of a combination of individual status, operating machinery and the operating environment. Individual status issues are mainly related to a poor physical state, poor mental state, or physical intelligence deficiency. Based on the characteristics of construction industry personnel, it is proposed that individual state problems are mainly manifested through poor physical and mental states and insufficient skill levels. There are many situations in which mechanical equipment is employed in construction at high altitudes, and there are potential unsafe factors related to the specification, configuration and proper operation of mechanical equipment. Therefore, the factors that influence machinery are mainly considered from the aspects of mechanical model configuration and operation standardization. According to the traditional HFACS framework, the factors that influence the working environment are related to the technical environment and the physical environment. In the technical environment, protection during work and personnel safety are emphasized. In the physical environment, surface cleanliness, good lighting and suitable weather will affect employee risk.

In summary, the preconditions for unsafe acts include the employee mental state (L2R1), physiological state (L2R2), and skill level (L2R3); mechanical equipment (L2R4); equipment operation and maintenance (L2R5); physical environment (L2R6); and technical environment (L2R7).

#### L3: Unsafe On-Site Supervision

Aksorn and Hadikusumo (37) verified the 16 key success factors identified in the safety literature and existing studies. The survey results showed that the factor that had the greatest impact on the safe production of construction projects was on-site safety management behavior. A construction site requires not only strict management but also timely inspection and acceptance, real-time assessments of the on-site conditions and employee behavior, and timely correction of unsafe phenomena (38). Supervisors of construction projects face severe penalties if regulations are violated, such as authorizing unqualified personnel to perform special operations and deliberately directing operators to perform dangerous work (39).

In summary, unsafe on-site supervision includes design work (L3R1), on-site management work (L3R2), safety inspection and acceptance (L3R3), and violations of regulations (L3R4).

#### L4: Adverse Organizational Influences

In the construction industry, it is necessary to rationally allocate relevant personnel, funds and supplies, and resource allocation has an important influence on organizational safety management (40). Yang and Fu (41) proposed that the internal safety culture of an enterprise will influence the behavior and attitude of employees through different channels and ultimately reduce the occurrence of safety accidents. Based on analyses of the factors that influence organizational success, strengthening process management at the enterprise level can ensure the smooth development of safe production activities. Additionally, decision-making and daily supervision at the organizational level can guide the scientific approach and standardized behavior at a project site.

In summary, the main adverse organizational influences include resource management (L4R1), safety culture (L4R2), and process management (L4R3).

#### L5: Adverse External Environment

Compared with the levels in the original HFACS framework, this level is new; it focuses on the influential factors outside an enterprise organization. Yang and Li (42) suggested that improving government supervision capabilities can effectively reduce the occurrence of construction safety accidents. Through expert consultation, it was found that external factors such as policy support, legal improvement, economic stability, and cultural penetration have a positive effect on construction safety.

In summary, the adverse external environment includes government supervision (L5R1) and the effects of politics, the economy, law and culture (L5R2).

### Manifestations of the Components of the Revised HFACS Framework

Based on a literature analysis and the opinions of 5 experts, the preliminary manifestations of the various components of the HFACS framework for construction falling accidents were sorted and revised. A total of 432 accident cases were utilized to assess the specific influence of each human-organizational factor. The open-loop analysis method was utilized to test the consistency of the description accuracy of the extracted factors, and expressions were obtained, as shown in Table 3.

**Table 3:** Components and manifestations of the HFACS framework for falling accidents.

**Level serial number**	**Level contents**	**Human factor**	**Manifestation**
First layer L1 (Phenomenon layer)	Unsafe acts	Perception and decision-making errors (L1R1)	A: The risk perception is inconsistent with the actual situation; B: Encountering problems beyond the scope of ability; C: The measures implemented to address the problem are incorrect; D: Employee safety awareness is limited
		Skill error (L1R2)	A: Insufficient safety skills and literacy; B: The method used in the implementation process is incorrect
		Operation violation (L1R3)	A: Habitual violations; B: Accidental violations
Second layer L2 (Influence layer)	Preconditions for unsafe acts	Poor mental state (L2R1)	A: There are random, habitual, and exploitative psychological behaviors, as well as the effects of being tired, in high office work; B: Inner pressure in daily work; C: Negative emotions in the process of getting along with co-workers
		Poor physiological condition (L2R2)	A: Overtired; B: Work with illness; C: Work after alcohol abuse; D: Physical impairment of hearing or vision
		Poor skill level (L2R3)	A: Insufficient construction experience; B: Lack of safety knowledge and safety training
		Mismatched work machine design (L2R4)	A: Safety warning label design for mechanical equipment is not obvious; B: Specifications and models of the equipment are inconsistent with the plan
		Irregular operation of equipment (L2R5)	A: The equipment is not used in strict accordance with the operation instructions; B: When the equipment fails, it is still used; C: The equipment is not regularly maintained
		Poor physical environment (L2R6)	A: Dirty, chaotic, and poor working environment; B: Inadequate lighting in the workplace; C: Limited working surface space
		Poor technical environment (L2R7)	A: Safety protection equipment is not utilized; B: No safety warning signs
Third layer L3 (Action layer)	Unsafe on-site supervision	Unreasonable design work (L3R1)	A: There is a lack of safety consideration in the design of the operation process; B: Too many tasks must be performed; C: Mismatched team members
		Loopholes in site management work (L3R2)	A: Safety rules and regulations are not implemented; B: Failure to quickly correct workers' incorrect behaviors; C: Failure to perform production safety management; D: Lack of timely and adequate technical disclosure
		Insufficient safety inspection and acceptance (L3R3)	A: Daily safety inspections are not performed; B: Insufficient investigations of hidden dangers; C: Lack of phased acceptance of a project
		Violation of regulations (L3R4)	A: Safety management personnel are not qualified to practice; B: Supervisors violate safety rules and regulations; C: False reporting and concealment of safety incidents
Fourth layer L4 (Root layer)	Adverse organizational influences	Inadequate resource management (L4R1)	A: Insufficient number of safety management personnel; B: Insufficient investment in safety production; C: Poor quality of purchased machinery and equipment
		Lack of safety culture (L4R2)	A: Management personnel do not pay attention to safety procedures; B: Lack of safety production regulations; C: Inadequate safety training
		Vulnerable process management (L4R3)	A: Lack of emergency plans; B: Unsound production safety responsibility system; C: Untimely work feedback; D: Inadequate safety precautions
Fifth layer L5 (Environmental layer)	Adverse external environment	Lax government supervision (L5R1)	A: Few on-site inspections by competent authorities; B: Insufficient punishment for illegal acts; C: Overlap of regulatory responsibilities and mutual prevarication in certain cases
		Influence of politics, economy, law and culture (L5R2)	A: Nonoptimal building safety laws and regulations; B: Unsound building safety policies and systems; C: Inadequate safety policy publicity; D: Overly formalized public supervision, public opinion supervision and social group supervision

## Analysis of the Influence of Human-Organizational Factors on Falling Accidents in Construction

### Bayesian Network Model Construction and Parameter Calculations

**Figure 3:**
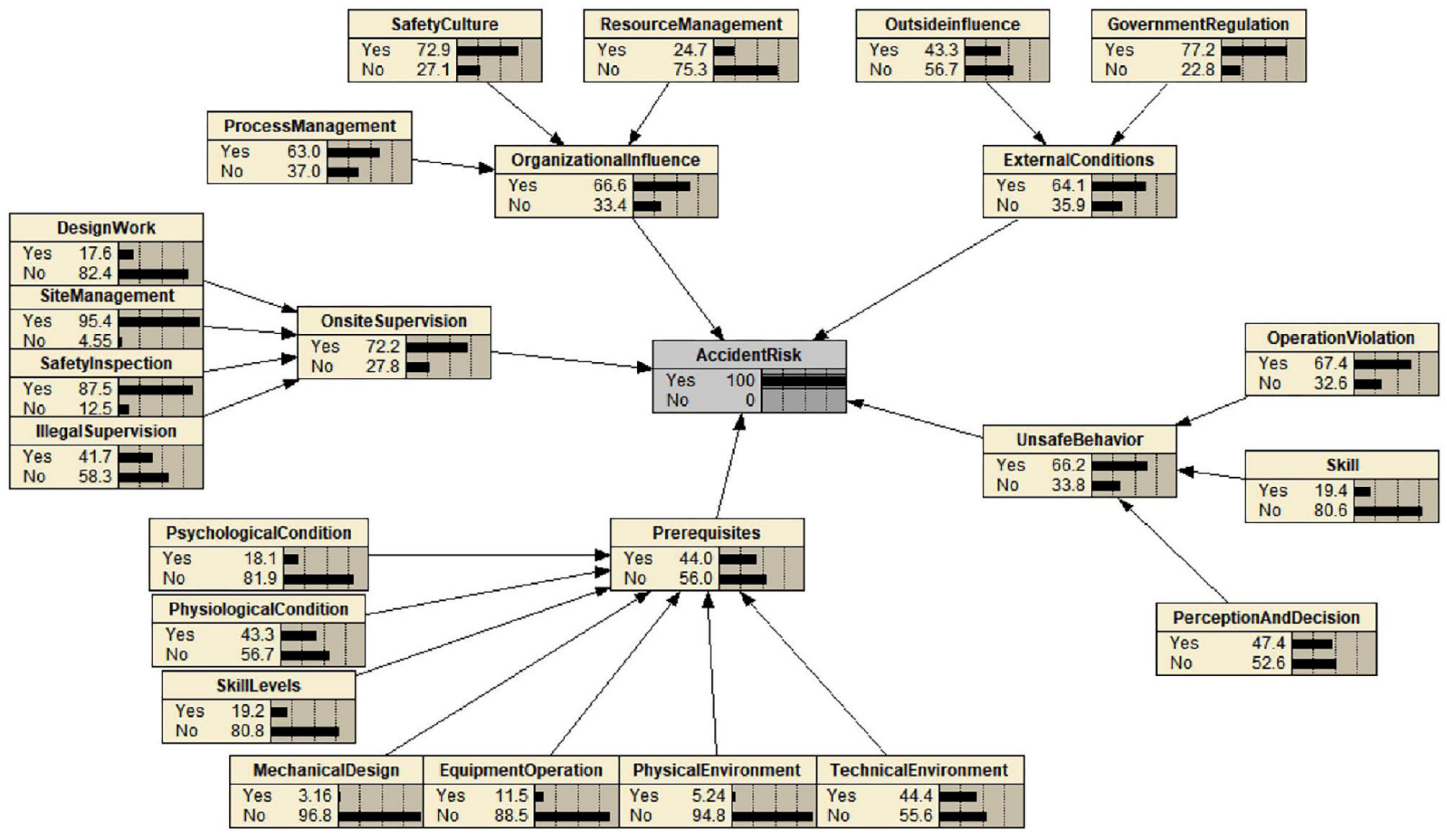
Reverse diagnosis and reasoning results of the BN for falling accidents.

Based on the revised HFACS model, the initial topological structure of the BN is constructed according to the dependence and independent relationships among factors. The model is adjusted in combination with the opinions of field experts to establish the human-organizational factor-BN risk assessment model for falling accidents in construction, as shown in Figure 3. According to the sample data obtained from the accident reports and fuzzy set theory, combined with Netica software and a Bayesian formula, the prior probabilities of root nodes and conditional probabilities of intermediate nodes in the network graph are calculated. Notably, there are two main methods for parameter estimation. For the nodes that have been fully reflected in the accident report, MLE is performed to obtain the BN parameter values. For the nodes that are not fully reflected in the accident report, fuzzy set theory is used to estimate the corresponding values. After sorting and coding the human-organizational factors involved in the falling accident reports, the parameter values for nodes L1R1, L1R2, L1R3, L2R3, L2R4, L2R5, L2R6, L2R7, L3R1, L3R2, L3R3, L4R1, L4R2, and L4R3 were calculated from accident reports, and the parameter values for nodes L2R1, L2R2, L5R1, and L5R2 were estimated with fuzzy set theory. The probability table of all nodes in the BN structure was obtained. Due to the limitation of content length, certain calculation details of the node parameters in BN are given in Appendix A.

### Bayesian Network Reasoning

#### Inference Analysis Process of the Bayesian Network

Under the condition that the BN topology and parameter values are determined, it is necessary to perform subsequent inference analysis on human-organizational factors of falling accidents of construction. The relevant data based on historical accident reports and expert consultations are collated and input into Netica for inference calculations, and the risk probability values at the nodes when a falling accident occurs are calculated. The calculation results are shown in Figure 3.

As shown in Figure 3, the following conclusions can be drawn when a falling accident occurs during construction.

1) As a five-layer defensive barrier for accident risks, unsafe on-site safety supervision (L3) has the greatest influence on the occurrence of falling accidents, followed by adverse organizational influence (L4), unsafe acts (L1), adverse external environment (L5), and preconditions for unsafe acts (L2). This result suggests that the on-site supervision of projects and the influence of the organization have a substantial roles in the occurrence of accidents. In addition, unsafe personal behavior has an important influence on risk prevention.

2) In the unsafe acts layer (L1), the number of falling accidents related to operation violations (L1R3) is the highest, accounting for 67.4% of all accidents, followed by perception and decision-making errors (L1R1), accounting for 47.5%. The number of accidents related to skill level errors (L1R2) is the lowest, accounting for 19.4% of all accidents. The results are consistent with those of Fogarty and Shaw in their study of unsafe behaviors: although the frequency of operational violations in daily work is much lower than the frequency of errors, the accident risk caused by violations is higher, and the potential harm is more serious.

3) In the preconditions of the unsafe acts layer (L2), the number of falling accidents related to the technical environment (L2R7) is the highest, accounting for 44.4% of all accidents, followed by poor physiological conditions (L2R2), which accounted for 43.3% of accidents. Poor skill level (L2R3), poor mental state (L2R1), irregular operation of equipment (L2R5), poor physical environment (L2R6), and mismatched work machine design (L2R4) had comparatively small impacts on the occurrence of accidents, accounting for 19.2, 18.1, 11.5, 5.24, and 3.16% of accidents, respectively. The results indicate that the occurrence of falling accidents in construction is closely related to construction safety protection measures and employee physiological factors; therefore, high-altitude operation requires rigorous and meticulous management of employees with appropriate climbing qualifications and the use of mechanical equipment.

4) In the unsafe on-site supervision layer (L3), the number of falling accidents related to loopholes in site management work (L3R2) is the highest, accounting for 95.4% of all accidents, followed by insufficient safety inspection and acceptance (L3R3), accounting for 87.5%. Violations of regulations (L3R4) and unreasonable design work (L3R1) factors were noted in only 41.7% and 17.6% of accidents, respectively. The results indicate that management and supervision factors at the field level have a decisive role in the occurrence of falling accidents, and the prevention of falling accidents should focus on these two factors.

5) In the adverse organizational influences layer (L4), the number of falling accidents related to the lack of safety culture (L4R2) is the highest, accounting for 72.9% of all accidents, followed by the vulnerable process management (L4R3) factor, accounting for 63% of all accidents. Inadequate resource management (L4R1) accounts for the smallest proportion of accidents at 24.7%. The results suggest that falling accidents are greatly affected by the safety culture of the enterprise, and cultural penetration and process management at the organizational level need to be considered by the internal management personnel of enterprises.

6) In the adverse external environment layer (L5), the number of falling accidents related to lax government supervision (L5R1) is the highest, accounting for 77.2% of all accidents, while the politics, the economy, law and culture (L5R2) factor accounts for a relatively small proportion of accidents at 43.3%. Government supervision problems occur not only in the preliminary bidding stage of a project but also in the acceptance stage, and the relevant law enforcement agencies must strictly comply with the management requirements throughout the project life cycle.

#### Sensitivity Analysis

**Figure 4:**
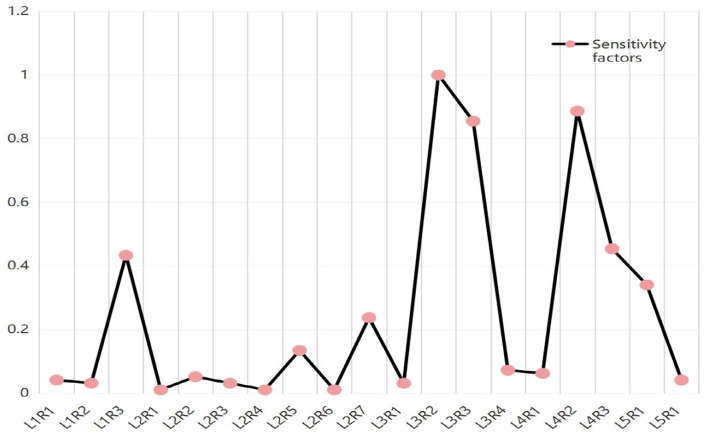
Sensitivity of human-organizational factors related to falling accidents.

With Netica software and relevant calculation formulas, the changes in target node probabilities caused by changes in parent node factors were quantified, and the key factors in the Bayesian risk assessment model were identified based on the measured sensitivity of human-organizational factors, as shown in Figure 4. Based on the calculation results in the figure, the sensitivity factors corresponding to loopholes in site management work, lack of safety culture, insufficient safety inspection and acceptance, vulnerable process management, and operation violations are relatively large, that is, these factors are the key causal factors of falling accidents in construction. It is necessary to focus on the management of these factors during risk identification and control at the human-organizational level to reduce the occurrence of falling accidents in construction.

## Conclusion

As the accident type accounting for the largest proportion of construction safety accidents, reducing the number of falling accidents by assessing the key causes and proposing control and defensive measures can effectively decrease financial and personnel losses. Due to the lack of adequate description of certain human factors, it is difficult to obtain an exact estimation of their occurrence possibility. Therefore, a BN risk analysis based on fuzzy theory under uncertainty is proposed to calculate the system reliability and identify the most sensitive factors of an accident. The final conclusions are presented as follows:

1) Based on the HFACS framework, characteristics of falling accidents in construction and existing literature, a revised HFACS framework for falling accidents was established. This framework encompasses the human-organizational factors that can cause falling accidents. A systematic structural framework including 5 layers (L1 phenomenon layer, L2 influence layer, L3 action layer, L4 root layer, and L5 environment layer) and 19 detailed causal factors was obtained to assess the causes of falling accidents.

2) With the BN inference analysis method, the revised HFACS framework was converted to a BN topology, and the probability value of each node in the network diagram was calculated based on the collected accident reports and fuzzy set theory. Through BN reasoning, the following results were obtained. In the five-layer falling accident prevention system, on-site safety supervision, organizational factors and unsafe acts of employees have vital roles in the occurrence of accidents. At the level of on-site safety supervision, lack of on-site management and insufficient safety inspection have the largest impacts on the occurrence of accidents. A lack of safety culture and vulnerable process management at the organizational factor level highly influence the organization and management of enterprises. At the level of unsafe acts, operation violations and perception and decision-making errors directly cause the occurrence of major falling accidents.

3) By combining BN reasoning with relevant formulas, a sensitivity factor analysis of the nodes in the BN risk assessment model was performed. The results indicate that when falling accidents occur, the most likely human-organizational factors are loopholes in site management work, lack of safety culture, insufficient safety inspection and acceptance, vulnerable process management, and operational violations. The calculated values of the corresponding sensitivity factors were relatively large, indicating that these factors are the key human-organizational factors of falling accidents.

4) Through sorting and coding of the collected accident reports, it was determined that management, inspection and education should be prioritized. Moreover, employee skill level, physiological state, psychological state, external politics, economy, law, and other factors had relatively minimal influence on falling accidents. Thus, in-depth investigations of specific scenarios are necessary. In addition, during the analysis of causal factors, illegal supervision behavior and a poor technical environment account for small proportions of the total number of falling accidents, but they have a considerable impact on project safety risks. Additionally, a single factor may create considerable safety hazards for a project, and influential factors should be fully considered by construction safety and risk management personnel.

In this paper, the combination of HFACS and BN is applied to the analysis of the impact of human-organizational factors of falling accidents in construction, which not only expands the application of BN in the field of safety but also helps to realize the quantitative analysis of accident human factors based on accident investigation data. However, this paper has limitations. BN reasoning based on historical accident texts is helpful for accident investigators to more accurately and comprehensively investigate the human-organizational factors that cause accidents. However, the existing accident investigation reports focus more on the description of accident responsibility identification, and the in-depth human factor investigation of the accident is insufficient. Therefore, it is necessary to rely on field experts in the process of risk identification and BN model construction. Our subsequent research objectives will focus on the collection of multisource data for security performance prediction and the use of more intelligent knowledge analysis technology to realize automatic knowledge management.

## Data Availability Statement

The original contributions presented in the study are included in the article/Supplementary Material, further inquiries can be directed to the corresponding author.

## Author Contributions

XL and QL contributed to conception and design of the study. ZQ organized the database. All authors contributed to manuscript revision, read, and approved the submitted version.

## Funding

The authors gratefully acknowledge the Fundamental Research Funds for the Central Universities (grant no. 2020ZDPYSK02).

## Conflict of Interest

The authors declare that the research was conducted in the absence of any commercial or financial relationships that could be construed as a potential conflict of interest.

## Publisher's Note

All claims expressed in this article are solely those of the authors and do not necessarily represent those of their affiliated organizations, or those of the publisher, the editors and the reviewers. Any product that may be evaluated in this article, or claim that may be made by its manufacturer, is not guaranteed or endorsed by the publisher.
