# Supplementary material for: The Influence of Human-Organizational Factors on Falling Accidents From Historical Text Data
Source: Front Public Health. 2022 Jan 11;9:783537. doi: 10.3389/fpubh.2021.783537 (PMC8787334; doi:10.3389/fpubh.2021.783537)
Supplement: Supplementary file 1 [file Table_1.DOC]

### Appendix A:

Table S1 The prior probability value of root node fully reflected in accident reports

| Root node | L1R1 | L1R2 | L1R3 | L2R3 | L2R4 | L2R5 | L2R6 | L2R7 |
| --- | --- | --- | --- | --- | --- | --- | --- | --- |
| State=Yes | 0.47 | 0.19 | 0.67 | 0.19 | 0.03 | 0.12 | 0.05 | 0.44 |
| State=No | 0.53 | 0.81 | 0.33 | 0.81 | 0.97 | 0.88 | 0.95 | 0.56 |
| Root node | L3R1 | L3R2 | L3R3 | L3R4 | L4R1 | L4R2 | L4R3 |  |
| State=Yes | 0.18 | 0.95 | 0.88 | 0.42 | 0.25 | 0.73 | 0.63 |  |
| State=No | 0.82 | 0.05 | 0.12 | 0.58 | 0.75 | 0.27 | 0.37 |  |

Table S2 Conditional probability distribution of node “Unsafe acts”

| L1R1 | Yes | | | | No | | | |
| --- | --- | --- | --- | --- | --- | --- | --- | --- |
| L1R2 | Yes | | No | | Yes | | No | |
| L1R3 | Yes | No | Yes | No | Yes | No | Yes | No |
| Yes | 0.923 | 0.237 | 0.739 | 0.318 | 0.716 | 0.396 | 0.634 | 0.368 |
| No | 0.077 | 0.763 | 0.261 | 0.682 | 0.284 | 0.604 | 0.366 | 0.632 |
